# Risk for cervical herniated intervertebral disc in dentists: a nationwide population-based study

**DOI:** 10.1186/s12891-019-2559-3

**Published:** 2019-05-04

**Authors:** Chien-Cheng Huang, Ping-Jang Kuo, Chien-Chin Hsu, Hung-Jung Lin, Shih-Bin Su, Jhi-Joung Wang, Shih-Feng Weng

**Affiliations:** 1 Department of Emergency Medicine, Chi-Mei Medical Center, 901 Zhonghua Road, Yongkang District, Tainan City, 710 Taiwan; 2 Department of Senior Services, Southern Taiwan University of Science and Technology, Tainan, Taiwan; 3 Department of Environmental and Occupational Health, College of Medicine, National Cheng Kung University, Tainan, Taiwan; 4 Department of Biotechnology, Southern Taiwan University of Science and Technology, Tainan, Taiwan; 5 Department of Emergency Medicine, Taipei Medical University, Taipei, Taiwan; 6 Department of Occupational Medicine, Chi-Mei Medical Center, Tainan, Taiwan; 7 Department of Leisure, Recreation and Tourism Management, Southern Taiwan University of Science and Technology, Tainan, Taiwan; 8 Department of Medical Research, Chi-Mei Medical Center, Tainan, Taiwan; 9 Department of Healthcare Administration and Medical Informatics, Kaohsiung Medical University, 100 Shin-Chuan 1st Road, Kaohsiung, 807 Taiwan; 10 Department of Medical Research, Kaohsiung Medical University Hospital, Kaohsiung, Taiwan

**Keywords:** Cervical, Dentist, Herniated intervertebral disc, Occupation, Prolonged static posture, Younger

## Abstract

**Background:**

Prolonged static postures (PSPs) may predispose dentists to develop cervical herniated intervertebral disc (C-HIVD); however, there is limited evidence supporting this in the literature thus far. We conducted this study to fit the data gap.

**Methods:**

We conducted a retrospective nationwide population-based study using the Taiwan National Health Insurance Research Database to identify 10,930 dentists, an identical number of age- and sex-matched participants from the general population, and 73,718 other health care providers (HCPs, non-dentists). Comparisons for the risk of developing C-HIVD between dentists and the general population, and between dentists and other HCPs were performed by tracing their medical histories between 2007 and 2011.

**Results:**

Dentists had a cumulative incidence rate of 1.1% for C-HIVD during the 5-year follow-up period. Overall, there was no difference of the risk for C-HIVD between dentists and the general population after adjusting for hypertension, hyperlipidemia, liver disease, mental disorders, diabetes mellitus, coronary artery disease, chronic obstructive pulmonary disease, malignancy, stroke, and renal disease (adjusted odds ratio [AOR]: 1.2, 95% confidence interval [CI]: 0.9–1.6). However, stratified analysis showed that younger dentists (≤ 34 years) had a trend of higher risk for C-HIVD than members of the younger general population (AOR: 1.9, 95% CI: 0.9–4.1). There was no difference found between dentists and other HCPs (AOR: 0.9, 95% CI: 0.8–1.1).

**Conclusion:**

Younger dentists had a trend of higher risk of developing C-HIVD than members of the general population.

## Background

Cervical herniated intervertebral disc (C-HIVD) is a common cause of compressive cervical radiculopathy, a pathologic process affecting the nerve root and thus causing neck, shoulder, or arm pain, sensory symptoms, weakness, or diminished deep tendon reflexes [1, 2]. Risk factors of C-HIVD are age, race, lack of regular exercise, tobacco use, and poor posture caused by engaging in activities such as holding prolonged static postures, lifting heavy objects, frequent platform diving, operating vibrating equipment, playing golf, and experiencing other types of physiological trauma [1–4]. The symptom of C-HIVD can be very disruptive, and the cumulative physiological damage can lead to an injury or a career-ending disability [5].

Prolonged static postures (PSPs) are often unavoidable in daily dental operations [4, 6]. Forward-head postures which involve holding the neck and head in an unbalanced forward position to gain better visibility during treatment are common among dentists [4, 6]. Sustained contraction of the cervical muscles due to the PSPs may cause the weakening of spinal discs, which in turn increases the risk of disc degeneration of herniation [4]. A previous study reported that orthopedists had a trend of higher risk for C-HIVD than other physician specialists [3]. The possible reason may be the PSPs of the orthopedists during the operations [3]. There were many studies reported that neck pain were very common and troublesome for their careers in the dentists [6–8]. Rahmani et al. reported that point, last month, last year and lifetime prevalence of neck pain were 19.3, 27.3, 29.9 and 34.7%, respectively [6]. Shaik et al. reported that 70% of dental surgeons experienced neck pain [7]. Dentists suffer a high prevalence of musculoskeletal injury [9]. However, the typical working hours and typical procedures that require prolonged forward-head postures in the dentists and comparison for neck pain between dentists and general population are still unclear. In addition, we did not find studies specific for C-HIVD, even after searching with keywords such as “cervical”, “herniated intervertebral disc”, “radiculopathy”, and “dentist” in PubMed and Google Scholar. Therefore, we conducted this study intending to clarify whether the risk for C-HIVD is higher in the dentists comparing to the general population and other health care providers (HCPs).

## Methods

### Data sources

This is a retrospective cohort study using a national database, the Taiwan National Health Insurance Research Database (NHIRD). The NHIRD contains registration files and original claims data including International Statistical Classification of Diseases (ICD) codes for reimbursement for approximately the entire population of Taiwan [10]. After data de-identification, the NHIRD is provided to the scientists in Taiwan for research purposes [10].

### Identification of dentists, general population, and other HCPs

**Fig. 1:**
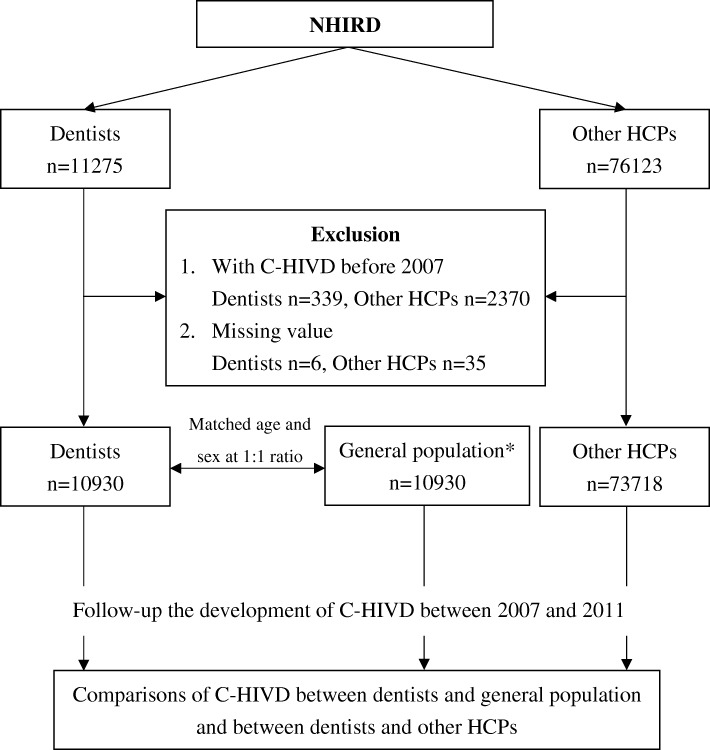
Flowchart of this study. NHIRD, National Health Research Database; C-HIVD, cervical herniated intervertebral disc; HCP, health care provider. *Exclusion of participants with C-HIVD before 2007 and missing data

We identified all the dentists from the 2009 registry for medical personnel in the NHIRD (Fig. 1) [11–14]. The 2009 registry for medical personnel we used included all the HCPs registered in 2009 and their medical histories between 1996 and 2012. An identical number of age- and sex-matched participants from the general population was identified for comparison. In order to decrease the number of potential confounders, we also identified a cohort consisting of other HCPs who may share similar working environments and socioeconomic status with the dentists for comparison [14–16]. The other HCPs included all the physicians, pharmacists, medical technicians, audiologists, consultant experts, clinical experts, dietitians, social workers, and language experts identified in the database we used, which were not matched with the dentists or general population. Age subgroups were categorized as ≤34, 35–59, and ≥ 60 years [14–17]. The underlying comorbidities were defined as hypertension (HTN) (ICD-9-CM code: 401–405), hyperlipidemia (ICD-9-CM code: 272), liver disease (ICD-9-CM code: 570–576), mental disorders (ICD-9-CM code: 290–319), diabetes mellitus (DM) (ICD-9-CM code 250), coronary artery disease (CAD) (ICD-9-CM code: 410–414), chronic obstructive pulmonary disease (COPD) (ICD-9-CM code: 496), malignancy (ICD-9-CM code: 140–208), stroke (ICD-9-CM code: 436–438), and renal disease (ICD-9-CM code: 580–593) [14]. The underlying comorbidities included in this study were based on having received the diagnosis during at least three ambulatory care visits, or at least one hospitalization [14, 15]. We included and adjusted for these underlying comorbidities in the logistic regression to control confounding effects [14, 18]. The participants who had been diagnosed with C-HIVD (ICD-9-CM codes: 722.0, 722.4, 722.71, or 722.91) before 2007 were excluded. C-HIVD was defined as the participants who received the diagnosis during at least one hospitalization or ambulatory care. We excluded the participants with C-HIVD before 2007 because we wanted to calculate the cumulative incidence of C-HIVD by following up between 2007 and 2011.

### Comparison of the risk of developing C-HIVD

We followed up the development of C-HIVD in the participants between 2007 and 2011 to compare the cumulative incidence of C-HIVD between dentists and general population, and between dentists and other HCPs. To assess whether age and sex were effect modifiers, stratified analyses for age and sex subgroups were also performed.

### Ethics statement

This study was approved by the Institutional Review Board at the Chi-Mei Medical Center. We conducted this study strictly according to the Declaration of Helsinki. This is a secondary data analysis article from the NHIRD and all necessary permissions were obtained to access and use the data. Informed consents from the participants are waived because the NHIRD contains de-identified information, which does not affect the rights and welfare of the participants.

### Statistical analysis

An independent *t*-test for continuous variables and a chi-square test for categorical variables were used in the comparison of demographic characteristics and underlying comorbidities between dentists and members of the general population. Conditional logistic regression analysis was performed to compare the risk of developing C-HIVD between dentists and general population by adjusting for HTN, hyperlipidemia, liver disease, mental disorders, DM, CAD, COPD, malignancy, stroke, and renal disease. Unconditional logistic regression was performed to compare the risk of developing C-HIVD between dentists and other HCPs by adjusting for age, sex, HTN, hyperlipidemia, liver disease, mental disorder, DM, CAD, COPD, malignancy, stroke, and renal disease. The 5-year (i.e., follow-up period between 2007 and 2011) cumulative incidences of C-HIVD in the dentists, general population, and other HCPs were also calculated. We used SAS 9.4 for Windows (SAS Institute, Cary, NC, USA) for all analyses. The significance level was set at 0.05 (two-tails).

## Results

Initially, we identified 11,275 dentists and 76,123 other HCPs from the NHIRD (Fig. 1). After excluding participants with C-HIVD before 2007 and missing data, 10,930 dentists, 10,930 participants from the general population, and 73,718 other HCPs were recruited for this study (Table 1). The rate of missing data in the dentists and other HCPs were 0.053% (6/11275) and 0.046% (35/76123), respectively. The mean ages (standard deviation) of the dentists, members of the general population, and other HCPs were 43.7 (11.3) years, 43.7 (11.3) years, and 42.5 (12.2) years. Male participants predominated in all the three cohorts. Dentists had lower prevalence of mental disorders, DM, COPD, malignancy, stroke, and renal disease, but higher prevalence of HTN and hyperlipidemia than the general population.

**Table 1:** Demographic characteristics and underlying comorbidities among dentists, general population, and other HCPs

	Dentists (*n* = 10,930)	General population (*n* = 10,930)	Other HCPs (*n* = 73,718)	*p*-value*
Age (years)	43.7 ± 11.3	43.7 ± 11.3	42.5 ± 12.2	> 0.999
Age (years)				
≤ 34	2943 (26.9)	2943 (26.9)	23,142 (31.4)	> 0.999
35–59	7092 (64.9)	7092 (64.9)	44,307 (60.1)	
≥ 60	895 (8.2)	895 (8.2)	6269 (8.5)	
Sex				
Male	8276 (75.7)	8276 (75.7)	45,739 (62.1)	
Female	2654 (24.3)	2654 (24.3)	27,979 (38.0)	> 0.999
Comorbidity				
HTN	1957 (17.9)	1757 (16.1)	12,132 (16.5)	< 0.001
Hyperlipidemia	1562 (14.3)	1236 (11.3)	10,208 (13.9)	< 0.001
Liver disease	1166 (10.7)	1101 (10.1)	7350 (10.0)	0.149
Mental disorder	898 (8.2)	1209 (11.1)	9033 (12.3)	< 0.001
DM	796 (7.3)	876 (8.0)	4440 (6.0)	0.042
CAD	581 (5.3)	523 (4.8)	3235 (4.4)	0.073
COPD	483 (4.4)	641 (5.9)	5203 (7.1)	< 0.001
Malignancy	240 (2.2)	308 (2.8)	1921 (2.6)	0.003
Stroke	197 (1.8)	295 (2.7)	1411 (1.9)	< 0.001
Renal disease	128 (1.2)	186 (1.7)	894 (1.2)	0.001

*Comparison between dentists and general population. Data are number (%) or mean ± SD. *HCP* health care provider, *HTN* hypertension, *DM* diabetes mellitus, *CAD* coronary artery disease, *COPD* chronic obstructive pulmonary disease

The 5-year cumulative incidences of C-HIVD in the dentists and members of the general population were 1.1 and 1.0%, respectively (Table 2). In the dentists, there was no difference of 5-year cumulative incidences of C-HIVD between two sexes. Conditional logistic regression analysis showed that the risk for C-HIVD was not different between dentists and general population (odds ratio [OR]: 1.1, 95% confidence interval [CI]: 0.9–1.5). The adjusted OR (AOR) (95% CI) was 1.2 (0.9–1.6) after adjusting for HTN, hyperlipidemia, liver disease, mental disorder, DM, CAD, COPD, malignancy, stroke, and renal disease. Stratified analysis showed that no difference existed among the sex subgroups. Dentists had a trend of higher risk for C-HIVD than the general population in the age of ≤34 years (AOR: 1.9, 95% CI: 0.9–4.1, *p*-value: 0.091). However, the differences were not significant in the age subgroups of 35–59 years (AOR: 1.2, 95% CI: 0.9–1.6) and ≥ 60 years (AOR: 0.7, 95% CI: 0.3–1.5). Comparisons of the risk for C-HIVD between dentists and other HCPs were not different in the overall (AOR: 0.9, 95% CI: 0.8–1.1) and stratified analyses by age and sex subgroups (Table 3).

**Table 2:** Comparison of the risk for C-HIVD between dentists and general population by conditional logistic regression

Variable	Number (%)	OR (95% CI)	AOR (95% CI)^a^	*p*-value†
Dentists (*n* = 10,930)	122 (1.1)	1.1 (0.9–1.5)	1.2 (0.9–1.6)	0.175
General population (n = 10,930)	107 (1.0)	reference	reference	
Sex subgroup				
Male				
Dentists (*n* = 8276)	94 (1.1)	1.1 (0.8–1.4)	1.1 (0.8–1.5)	0.385
General population (n = 8276)	87 (1.1)	reference	reference	
Female				
Dentists (*n* = 2654)	28 (1.1)	1.4 (0.8–2.5)	1.5 (0.8–2.6)	0.204
General population (*n* = 2654)	20 (0.8)	reference	reference	
Age subgroup				
≤ 34 years				
Dentists (*n* = 2943)	19 (0.7)	1.7 (0.8–3.6)	1.9 (0.9–4.1)	0.091
General population (*n* = 2943)	11 (0.4)	reference	reference	
35–59 years				
Dentists (*n* = 7092)	92 (1.3)	1.1 (0.8–1.5)	1.2 (0.9–1.6)	0.249
General population (*n* = 7092)	81 (1.1)	reference	reference	
≥ 60 years				
Dentists (*n* = 895)	11 (1.2)	0.7 (0.3–1.6)	0.7 (0.3–1.5)	0.319
General population (*n* = 895)	15 (1.7)	reference	reference	

C-HIVD cervical herniated intervertebral disc, *OR* odds ratio, *AOR* adjusted odds ratio, *CI* confidence interval, *HTN* hypertension, *DM* diabetes mellitus, *CAD* coronary artery disease, *COPD* chronic obstructive pulmonary disease. ^a^Adjusted for HTN, hyperlipidemia, liver disease, mental disorder, DM, CAD, COPD, malignancy, stroke, and renal disease. †For AOR

**Table 3:** Comparison of the risk for C-HIVD between dentists and other HCPs by unconditional logistic regression

Variable	Number (%)	OR (95% CI)	AOR (95% CI)^a^	*p*-value†
Dentists (*n* = 10,930)	122 (1.1)	0.9 (0.7–1.1)	0.9 (0.8–1.1)	0.558
Other HCPs (*n* = 73,718)	911 (1.2)	reference	reference	
Sex subgroup				
Male				
Dentists (*n* = 8276)	94 (1.1)	0.9 (0.7–1.1)	0.9 (0.7–1.2)	0.542
Other HCPs (*n* = 45,739)	583 (1.3)	reference	reference	
Female				
Dentists (*n* = 2654)	28 (1.1)	0.9 (0.6–1.3)	0.9 (0.7–1.4)	0.893
Other HCPs (*n* = 27,979)	328 (1.2)	reference	reference	
Age subgroup				
≤ 34 years				
Dentists (*n* = 2943)	19 (0.7)	1.1 (0.7–1.8)	1.2 (0.7–2.0)	0.444
Other HCPs (*n* = 23,142)	135 (0.6)	reference	reference	
35–59 years				
Dentists (*n* = 7092)	92 (1.3)	0.8 (0.7–1.0)	0.9 (0.7–1.1)	0.402
Other HCPs (*n* = 44,307)	682 (1.5)	reference	reference	
≥ 60 years				
Dentists (*n* = 895)	11 (1.2)	0.8 (0.4–1.5)	0.9 (0.5–1.6)	0.618
Other HCPs (*n* = 6269)	94 (1.5)	reference	reference	

*C-HIVD* cervical herniated intervertebral disc, *HCP* health care provider, *OR* odds ratio, *AOR* adjusted odds ratio, *CI* confidence interval, *HTN* hypertension, *DM* diabetes mellitus, *CAD* coronary artery disease, *COPD* chronic obstructive pulmonary disease. ^a^Adjusted for age, sex, HTN, hyperlipidemia, liver disease, mental disorder, DM, CAD, COPD, malignancy, stroke, and renal disease. †For AOR

## Discussion

This study showed that 5-year cumulative incidence of the dentists was 1.1% without sex difference. Overall, the risk for C-HIVD was not different between dentists and the general population and between dentists and other HCPs. However, younger dentists (≤ 34 years) had a trend of higher risk for C-HIVD than the younger members of the general population. The increased risk was not found in the older dentists. There was no difference between dentists and other HCPs.

The trend of increased risk was only found in the younger dentists. The possible explanation is that younger dentists may have less knowledge and experience in preventing and managing work-related musculoskeletal problems than older dentists [19, 20]. Dentists often need to maintain static postures which require more than half of the body’s muscles to contract to hold the body motionless while resisting gravity [5]. These PSPs are much more taxing than moving forces [5]. If the body suffers from PSPs, the subsequent consequences may result: (1) muscle fatigue, (2) muscle imbalance, (3) muscle ischemia/necrosis, (4) trigger points and muscle substitution, (5) pain, (6) protective muscle contraction, (7) joint immobilization, (8) nerve compression, and finally (9) spinal disc degeneration or herniation [5].

Maintaining the cervical lordosis in the proper position is very important to prevent C-HIVD. However, postures which involve holding the neck and head in a forward position during dental operations often disrupt the balance of the neck [4]. In maintaining these poor postures, the cervical and thoracic muscles must contract constantly to support the head in the forward position, and this results in the vertebrae no longer being able to properly support the spine [4]. Subsequently, tension neck syndrome may develop, which includes symptoms such as headache, and chronic neck, shoulder, interscapular muscles, and arm pains [4]. Persistent contraction of cervical muscles may cause degeneration or herniation of the spinal discs [4]. The suggested strategies for prevention of C-HIVD include the strengthening and frequent relaxing and stretching of the neck muscles and preservation of the cervical lordosis by engaging in proper posture for all activities [4].

The trend of increased risk for C-HIVD in the younger dentists may impact their jobs. Dentists should receive education about musculoskeletal health, injury prevention, and dental ergonomics as early as possible [4]. In a review article, the authors suggested that most dentists lack the skills and knowledge for practicing in a manner that is ergonomically correct and the insufficiency of training may be due to lack of better teachers and teaching tools [4]. The following approaches could be adopted, including education of prevention, selection of ergonomic equipment, strategies for posture and position, and frequent stretching and strengthening exercises during breaks [4].

This study has the major strength of delineating an unclear issue through a nationwide population-based design. The limitations are as follows. First, the follow-up period of 5 years may be not enough to reflect the real difference in the comparisons due to the limitation of the data we used. Longer follow-up period may be needed to validate the current finding. Second, occupational exposures including postures during work, working hours, time spent in forward-head postures, personal life styles (e.g., exercise and habit), and tobacco use were not available in the NHIRD, and these factors may affect the causal relationship between exposure and disease. However, we included the underlying comorbidities such as HTN, DM, hyperlipidemia, and COPD for adjustment in the analyses, which may serve as surrogates for personal lifestyles and tobacco use. Third, dentists may treat themselves without seeking medical advice, and therefore using claims data for the analyses may underestimate the risk for C-HIVD in this population. Fourth, although this is a nationwide study, the result may not be generalized to other nations due to the differences in races, working environments, health insurances, and culture.

## Conclusion

This nationwide population-based study delineated that younger dentists had a trend of higher risk for C-HIVD than their younger counterparts from the general population. However, the same phenomenon was not identified in older dentists. Education on how to prevent and manage work-related musculoskeletal problems are suggested for this population of younger or more inexperienced dentists.

## Abbreviations

AOR: Adjusted odds ratio
CAD: Coronary artery disease
C-HIVD: Cervical herniated intervertebral disc
CI: Confidence interval
COPD: Chronic obstructive pulmonary disease
DM: Diabetes mellitus
HCPs: Health care providers
HTN: Hypertension
NHIRD: National Health Insurance Research Database
OR: Odds ratio
PSPs: Prolonged static postures
